# Model to Track Wild Birds for Avian Influenza by Means of Population Dynamics and Surveillance Information

**DOI:** 10.1371/journal.pone.0044354

**Published:** 2012-08-30

**Authors:** Anna Alba, Dominique J. Bicout, Francesc Vidal, Antoni Curcó, Alberto Allepuz, Sebastián Napp, Ignacio García-Bocanegra, Taiana Costa, Jordi Casal

**Affiliations:** 1 Centre de Recerca en Sanitat Animal, Universitat Autònoma de Barcelona-IRTA, Campus de la Universitat Autònoma de Barcelona, Barcelona, Spain; 2 Unité BioMathématiques et Epidémiologie – Environnement et Prédiction de la Santé des Populations TIMC, Centre national de la recherche scientifique. VetAgro Sup, Marcy l’Etoile, France; 3 Parc Natural del Delta de l’Ebre, Departament de Medi Ambient i Habitatge, Deltebre, Tarragona, Spain; 4 Departament de Sanitat i Anatomia Animals, Universitat Autònoma de Barcelona, Barcelona, Spain; 5 Departamento de Sanidad Animal, Facultad de Veterinaria, Universidad de Córdoba, Córdoba, Spain; University of Georgia, United States of America

## Abstract

Design, sampling and data interpretation constitute an important challenge for wildlife surveillance of avian influenza viruses (AIV). The aim of this study was to construct a model to improve and enhance identification in both different periods and locations of avian species likely at high risk of contact with AIV in a specific wetland. This study presents an individual-based stochastic model for the Ebre Delta as an example of this appliance. Based on the Monte-Carlo method, the model simulates the dynamics of the spread of AIV among wild birds in a natural park following introduction of an infected bird. Data on wild bird species population, apparent AIV prevalence recorded in wild birds during the period of study, and ecological information on factors such as behaviour, contact rates or patterns of movements of waterfowl were incorporated as inputs of the model. From these inputs, the model predicted those species that would introduce most of AIV in different periods and those species and areas that would be at high risk as a consequence of the spread of these AIV incursions. This method can serve as a complementary tool to previous studies to optimize the allocation of the limited AI surveillance resources in a local complex ecosystem. However, this study indicates that in order to predict the evolution of the spread of AIV at the local scale, there is a need for further research on the identification of host factors involved in the interspecies transmission of AIV.

## Introduction

Avian influenza (AI) is a dynamically evolving disease caused by highly contagious viruses, which affect a wide variety of avian and mammalian species, including humans. Numerous studies have focused on improving knowledge of AI ecology in natural reservoirs and hosts. Many scientific publications have documented the importance of wild aquatic birds as natural reservoirs of avian influenza viruses (AIV), especially Anseriformes and Charadriiformes [1]–[5]. Some of these studies have also evidenced that AIV may persist in the environment for extended periods under favourable conditions, and both continuous bird-to-bird and waterborne transmission are considered to be the most frequent modes of transmission and maintenance of AI infection in wild birds [4]–[9]. However, despite the improving insight into AI epidemiology achieved in recent years, much remains unknown with regard to AIV transmission. The complexity of AIV transmission is derived from the multiple interactions between different subtypes of AIV and hosts, the co-evolution between them, and the influence of the environment [10]. The need for global collation of existing wild bird AIV data and infrastructure, as well as the pooling of multidisciplinary expertise with different approaches and resources, has been highlighted [11]. Since the emergence and spread of H5N1 highly pathogenic avian influenza (HPAI) in both wild and domestic birds throughout Eurasia and Africa, AI has constituted an issue of major concern for public and animal health authorities around the world. Since 2005, the implementation of AI surveillance systems in wild birds has been compulsory in all European Member States. These systems were extended and harmonized on a European scale with the aim of early detection of H5N1 HPAI infection, the identification of possible carriers or intermediate risk species, and the collection of baseline information on the circulation of low pathogenic avian influenza viruses (LPAIV) in wild birds. Between 2006 and 2009, most of these programs included both active and passive surveillance. The selection of species for sampling had to be adapted to the avian population in each region, including the predominant species and population sizes, seasonality of migration patterns, migratory flyways, and mixing of species in particular habitats [11]–[13].

The purpose of this study was to construct a complementary tool to track those avian subpopulations at high risk of contacting any AI subtype at a given location and point in time, according to avian population dynamics and AIV occurrence.

## Materials and Methods

The model was based on the Monte-Carlo method and integrated information about the distribution, behaviour and affinity patterns of waterfowl populations living in this wetland, as well as surveillance data reported in Europe on the prevalence of AIV in migrant birds that may act as introducers within the period of study.

### Geographical Area of Study

The model was developed for the Ebre Delta, which is the main wetland in Catalonia (North-Eastern Spain), and one of the most important wetlands in the Mediterranean Littoral. The Ebre Delta is an area of 320 km^2^, consisting of multiple ecosystems, including lagoons of varying salinity and depth, sand dunes, salt marshes and rice fields with many resting sites for migratory waterfowl. In the wetland, the waterfowl population lives close to many commercial poultry holdings and backyard flocks of different species (chickens, ducks, turkeys, etc.). This area lodged a total of 235 bird species with a spring and summer population of around 100,000 waterfowl, and an autumn and winter population of 200,000 [14]. The surveillance carried out between July 2006 and June 2009 demonstrated the circulation of different strains of AIV among the wild bird population in this area [15]. Due to the abundance of waterfowl and the potential transmission of AIV from wild birds to domestic poultry, the Ebre Delta has been considered a high-risk area for the introduction of AIV from wild birds [16].

### Wild Bird Population

One of the inputs of the model was the average waterfowl population (P) of the Ebre Delta, based on the data recorded between 2001 and 2006 (official source DMAIH, 2007), in two different periods: autumn/winter, from October to February (Pw), and spring/summer, from March to September (Pb). Although several factors, such as the number of hatch-year birds during the spring and summer seasons, migratory movements, climate or hunting, may influence avian population size, given the uncertainty and variability of these factors, we assumed a closed avian population without changes of demography (no births, deaths, or migration) equal to P (P = Pw, Pb). P remained constant throughout each period studied and consisted of
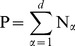
(1)


Where N_α_ was the number of birds of species α (α = 1, 2, …, d).These species were classified into two groups according to their susceptibility to AIV infection: species at high risk (α^R^) that could act as introducers and important disseminators of AIV, and intermediate risk species (α^B^) that were not considered important disseminators but might transport AIV from wildlife to poultry and vice-versa.

The criteria used to classify a species as α^R^ was: 1. This species showed migratory behaviour, 2. Its importance as a natural reservoir was previously documented in scientific studies [5], [17]–[18], and 3. The apparent AI prevalence (Prev_α_) reported by surveillance programs for AI in Europe in 2006/07 was higher than the apparent prevalence detected in other species for which their importance as natural reservoirs remained unknown [19]–[20].

Taking into account the AI occurrence reported in Europe during this period, the α^R^ group was composed mainly of dabbling ducks and terns. The remaining aquatic species that were abundant in the delta (census >50 individuals) and had a high degree of affinity with α^R^ were included as α^B^ (Table 1).

**Figure 1:**
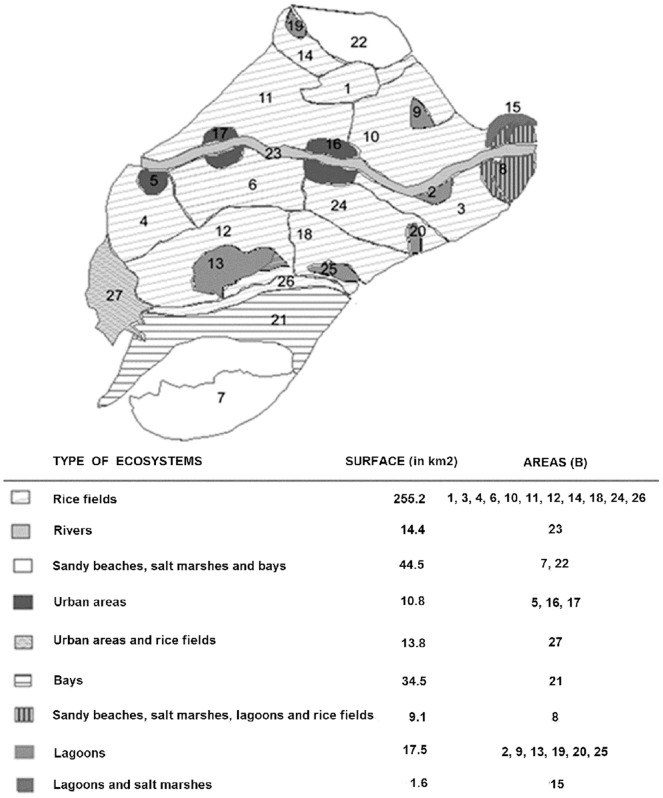
Type of ecosystems in the Ebre Delta and division into areas based on ecological and ornithological criteria.

Waterfowl are not homogeneously distributed in the Delta due to such factors as food availability, nesting sites and the presence of natural competitors or predators. Thus, based on ornithological and ecological criteria, the Ebre Delta Natural Park was subdivided into 27 areas designated as B, where B = 1, 2, …, 27 (Figure 1).

Each of these areas (B) contained a given population of birds of species α i.e. n_B,α_ such that:
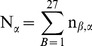
(2)


Where, N_α_ represents the total number of birds of α species in the Ebre Delta.

### Model Dynamics

The model simulated how an infectious migrant bird belonging to a species in the α^R^ group arrived in the Ebre Delta at the beginning of each period of study, and then represented the transmission dynamics over weekly intervals. The infected bird might infect other susceptible birds, which would consequently become infectious. These infected birds would return to the susceptible class after the infectious state. This susceptible–infectious-susceptible approach was adopted because of the lack of data to quantify immunity to AIV in the different avian species. Each bird “i” of species α might occupy two states with respect to AI: Iα_,i_ = 0, for susceptible, and Iα,_i_ = 1, for infectious. The dynamics of the infection were given by:
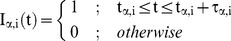
(3)


Where t_α,i_ was the date of the onset of infection for the bird “i” of species α and τ_α,i_, was the infectious period (or virus excretion period). Given the lack of species-specific data, a similar infectious period, based on previous studies [21]–[25], was assumed for all species. The duration of τ_α,i_ was represented by a normal distribution N(τ_α_, *σ* ^2^) with a mean τ_α,i_ = 2 weeks and a variance *σ* ^2^ = 1 week. For the primary case, a period δ which corresponded to the time passed between the infection of the bird and its arrival in the Delta, was discounted. The value of δ was obtained from a uniform distribution U[0, τ_α,i_ ].

**Table 1:** Information included in the model in relation to the species and censuses, classification of the risk group, and apparent prevalence [19], [20].

Family	Species (α)	Group of risk	Pw	Pb	Prev_α_
*Anatidae*	Pintail *(Anas acuta )*	High	1807	0	3.3%
	Shoveler *(Anas clypeata )*	High	11455	12	5.9%
	Teal *(Anas crecca )*	High	11262	0	4.3%
	Wigeon *(Anas penelope)*	Intermediate	2242	0	1.1%
	Mallard *(Anas platyrhynchos)*	High	42332	22062	4.2%
	Gadwall *(Anas strepera)*	Intermediate	2797	718	1.5%
	Greylag Goose *(Anser anser)*	Intermediate	840	0	0.3%
	Pochard *(Aythya ferina)*	High	523	6	4.2%
	Tufted Duck *(Aythya fuligula)*	High	55	0	8.3%
	Red-crested Pochard *(Netta rufina)*	Intermediate	3670	4412	0.9%
	Shelduck *(Tadorna tadorna)*	High	10074	204	3.5%
*Ardeidae*	Grey heron *(Ardea cinerea)*	Intermediate	2479	84	0.3%
*Charadriidae*	Kentish Plover *(Charadrius alexandrinus)*	Intermediate	735	884	0.0%
	Grey Plover *(Pluvialis squatarola)*	Intermediate	1455	0	1.2%
	Lapwing *(Vanellus vanellus)*	Intermediate	14280	0	0.2%
*Glareolidae*	Collared Pratincole *(Glareola pratincola)*	Intermediate	0	217	0.0%
*Laridae*	Audouin’s Gull *(Larus audouinii)*	Intermediate	94	20227	0.0%
	Slender-billed Gull *(Larus genei)*	Intermediate	251	1094	No data
	Herring Gull *(Larus michahellis)*	Intermediate	14850	12482	1.8%
	Black-headed Gull *(Larus ridibundus)*	Intermediate	50897	8016	1.1%
*Phoenicopteridae*	Greater Flamingo *(Phoenicopterus ruber)*	Intermediate	6970	1837	1.9%
*Podicipedidae*	Great Crested Grebe *(Podiceps cristatus)*	High	593	189	3.3%
	Little Grebe *(Tachybaptus ruficollis)*	High	620	620	4.8%
*Rallidae*	Coot *(Fulica atra)*	Intermediate	19595	9070	0.8%
*Recurvirostridae*	Black-winged Stilt *(Himantopus himantopus)*	Intermediate	5	3056	No data
	Avocet *(Recurvirostra avosetta)*	Intermediate	918	917	0.0%
*Scolopacidae*	Black-tailed Godwit *(Limosa limosa)*	Intermediate	6964	0	0.0%
	Ruff *(Philomachus pugnax)*	Intermediate	937	0	0.0%
	Redschnak *(Tringa totanus)*	Intermediate	1421	262	0.0%
*Sternidae*	Whiskered Tern *(Chlidonias hybridus)*	Intermediate	236	3016	No data
	Little Tern *(Sterna albifrons)*	Intermediate	0	681	No data
	Common Tern *(Sterna hirundo)*	High	0	8447	4.6%
	Gud-billed Tern *(Sterna nilotica)*	Intermediate	0	922	No data
	Sandwich Tern *(Sterna sandvicensis)*	Intermediate	0	4063	0.0%
**Total Census**			**210357**	**103504**	

Pw: population in the autumn and winter; Pb: population in the spring and summer; Prev_α_: apparent AI prevalence.

The probability of a α^R^ species acting as an AIV introducer (P_α_^R^) was dependent on the total census of α^R^ species (N_α_^R^) and its apparent prevalence *(*Prev*_α_* ^R^) detected between 2006 and 2007 (eq. 4). A multinomial distribution was assigned to define the probability of each α^R^ species, and the species that acted as the primary case was selected using the Monte-Carlo method.
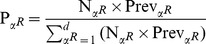
(4)


Once the α^R*^ species of the primary case was determined, the model simulated the area (B*) in which this infectious bird *(*I_α,i_ = 1) arrived, which was also determined using the Monte-Carlo method. A multinomial distribution was used to model the probability of the primary case arriving in each of these B areas (p_B_). This probability (p_B_) was dependent on the population of the species that acted as the primary case (α^R*^) in the different areas (n _B,α_^R*^ ).
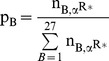
(5)


Given the lack of data on the number of contacts per week, the infectious period, and the transmission probability for each avian species, the number of secondary cases arising from an infected bird, i.e. the effective reproductive rate (R), could not be determined.

Therefore, to model the progress of disease, a scenario-approach was adopted. In this process, the primary case may infect birds belonging to the same species as the primary case or to a different species. It was assumed that an infectious bird was more likely to infect a bird of the same species than of a different species. Subsequently, the secondary cases might infect other birds (of the same or of a different species), and so on. Whether the disease progressed or not depended on the probability of an infectious bird infecting a bird of the same species and a bird of a different species. These probabilities were assumed to be higher for infected birds belonging to a risk species than to intermediate species.

In each study period, two hypothetic scenarios were simulated. In these scenarios different forces of infection were assumed. The values for the probabilities of infection of birds of the same and of different species for both risk and intermediate species in the two scenarios are presented in Table S1.In accordance with these values the number of secondary cases from each infected bird could take a value of 0, 1 or 2.

The model was run with 10,000 iterations for two scenarios within each period of study: 1. A self-extinguished epidemic with a low force infection resulting in R <1, and 2. An epidemic with a higher force infection resulting in R between 1 and 2 (Table S1).

Subsequently, the model determined the species of secondary cases based on the size of the population of the species different to α* included into the affected area (B*), and the degree of affinity between these species and the infected α* species.

The probability of an α species different to α* being infected by an infected α* bird in the B*area (p_α_ ) was given by the following multinomial distribution:
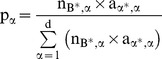
(6)


In which n_B*,α_ corresponded to the number of birds of α species different to α* in the B* area, and a_α*,α_ was the degree of affinity between these species and the infected α* species.

To determine the degree of affinity between species in different periods, an expert opinion session was held. The participants in this day session were four ornithologists with broad expertise in bird monitoring tasks in the area of study, and two veterinary epidemiologists, who designed the queries and moderated the session. The ornithologists were previously informed and a checklist was facilitated to quantify such different aspects as the probability of sharing habitat, feeding or nesting group, and gregarious behaviour. The query aspects were in accordance with the preliminary assessment proposed by Atkinson et al. [26] related to ornithological data relevant to the spread of AI. The experts answered the questionnaires in turn and were then provided with a summary of their forecasts. We encouraged them to revise their earlier answers in light of their replies, and after they had finally come to a consensus, we determined a score to assign the values that would serve as inputs of the model. Based on these data a probability of direct and indirect contact between different species was assigned (Table S2).

### Bird Movements in the Area

Initially, the model simulated that each infected bird was located at a specific point designated r_α,i_ =  (x_α,i_, y_α,i_) within the B* area. Assuming that the bird population was homogeneously distributed in each B* area, the r_α,i_ point was randomly determined. From its reference r_α,i_ location, an infected bird might move around the Ebre Delta following two patterns: local dispersion and long range dispersion. The probability of local movement or long range dispersion was given by φ_α,i_. Due to the lack of contrasted data, we assumed that this probability of movement (φ_α,i_) was equal to 0.5 for all species. In the new location, the infected bird may transmit AIV to susceptible birds.

The local dispersion was defined by an exponential distribution of mean 200 meters from the fixed reference r_α,i_ location. In the event of long range dispersion, the distances of dispersion (ρ_α_) of the infected bird were simulated considering the patterns of movement for each species in each period. Different ranges of long range dispersion were defined with the help of ornithologists (between 200 meters and 1 km, between 1 and 2 km, between 2 and 5 km, and more than 10 km). Then, for each species and each period, the probabilities of movement at such distances were also defined based on the opinion of experts (Table S3). The direction of these movements was randomly defined, discarding movements to the sea.

### Transmission Dynamics

**Figure 2:**
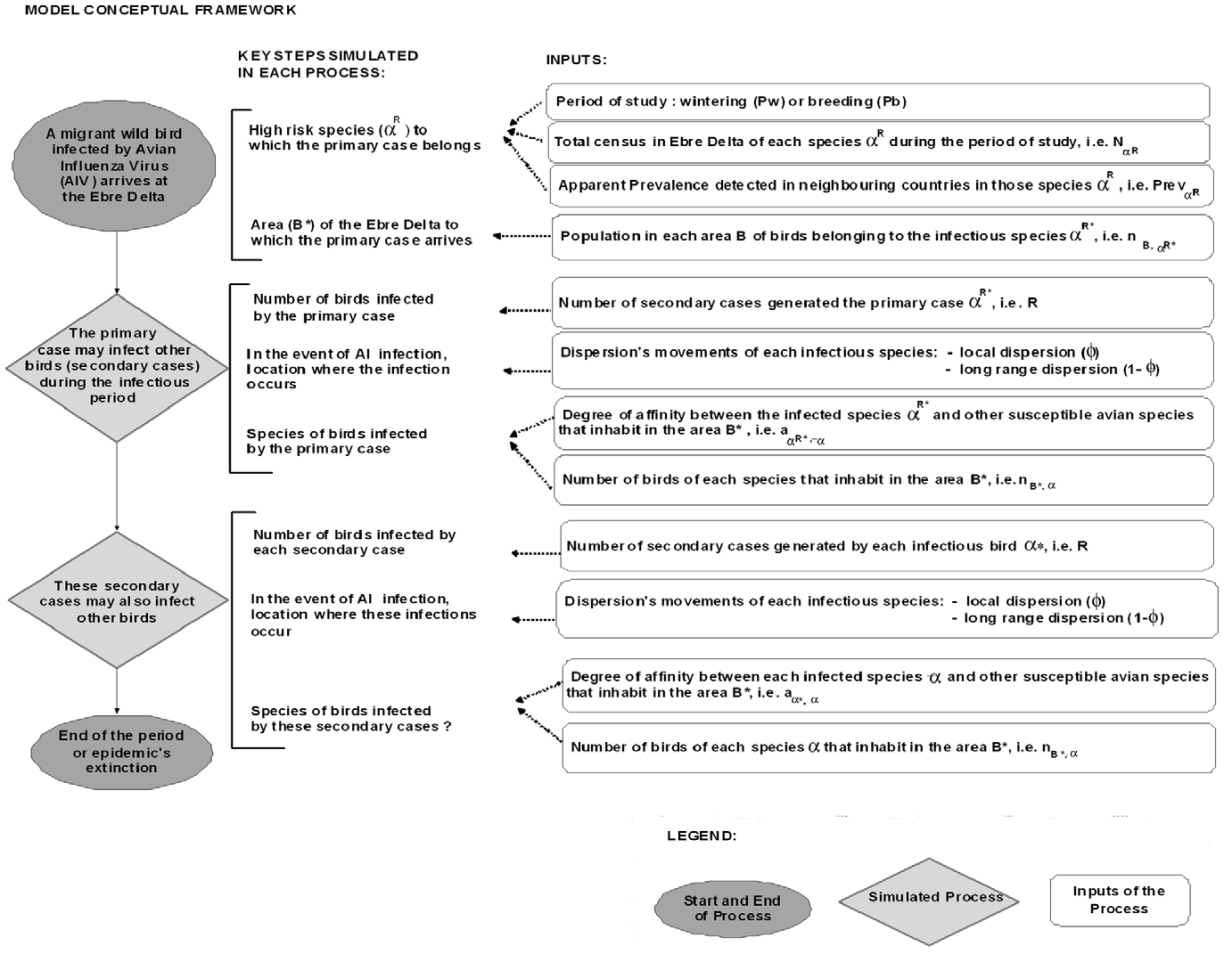
Flowchart of the processes simulated by the model.

The newly infected birds continued the same process of transmission dynamics throughout the period simulated and could infect new susceptible birds, I_α,i_ = 0. Each iteration starts with the introduction of an infected bird, i.e. primary case, which might infect susceptible birds, i.e. secondary cases, and the transmission continued until the elimination of the infection or the end of the period of study (Table S4 and Figure 2).

We assumed that during the spring and summer period the infected bird arrived in the delta in the first week of March, and the spread of AIV might occur during the next 31 weeks. Whereas, during the autumn and winter period the infectious bird arrived in the first week of October and the spread of AIV might occur during the following 21 weeks. Rather than specifically dealing with all birds in the study area, the simulations only kept track of infected birds.

### Software

The model was constructed in Microsoft Visual Basic 6.0. The division of the different areas of the Ebre Delta was generated using new shape files created with ArcCatalog (ESRI^®^, Redlands, CA, USA) from a raster map. The input and output data were stored in Microsoft Office Access 2003. For the analysis and representation of the outputs, SPSS 15.0 for Windows (SPSS Inc., Chicago, Illinois) and ArcGIS.9 (ESRI, Redlands, CA, USA) were used.

### Outputs Obtained

For each scenario, the model provided the following estimates: most likely species to introduce AIV and most likely sites affected by these incursions, and species and areas most likely affected by these contacts.

### Sensitivity Analysis

A sensitivity analysis was conducted to estimate the influence of different inputs on the results. The inputs assessed were: 1. Initial apparent prevalence of high-risk species, 2. Duration of the infectious period, 3. Values of affinity, 4. Movement distances, 5. Probability of movement at local or long range distance, and 6. Average number of secondary cases.

This analysis consisted of running trial simulations while varying the main inputs across an 80% range of the initial value or distribution. For example, the initial prevalence considered for Mallards (*Anas platyrhynchos)* was 0.3, in the original model, and this value varied between 0.06 and 0.54 in the sensitivity analysis.

For each scenario, 10,000 iterations were run with the original input values and the 80% range variation in each of the inputs assessed. The medians of outputs were compared using a Mann-Whitney test and represented in a box plot graph.

## Results

The model predicted that during the spring and summer most AIV would be introduced by Mallards (in 68% of cases) and Common Terns *(Sterna hirundo)* (29%), followed by 3% for other species belonging to the *Anatidae* or *Podicipedidae* families. Whereas, in the autumn and winter, the model estimated that most AIV would be introduced by different species of wild duck (89%), such as Mallards (57%), Shovelers *(Anas clypeata)* (21%) and Common Teals (*Anas crecca*) (16%), followed by 6% for other species of *Anatidae* or *Podicipedidae* families such as Great Crested Grebes (*Podiceps cristatus*).

Secondly, the model provided information about those species that would be at high risk as consequence of the spread of these AIV incursions. The results indicated that if in spring and summer the primary cases were Mallards, the species most likely to be affected would also be Mallards, as well as Coots (*Fulica atra*) and Black-headed Gulls (*Larus ridibundus*). However, if the introducers were Common Terns, the species most likely to be affected would be other Common Terns and Sandwich Terns (*Sterna sandvicensis*). In the autumn and winter, with the AIV incursion from different dabbling ducks, the species at highest risk would be Mallards, Coots, Herring Gulls (*Larus michahellis*), Shovelers, Common Teals, Pintails (*Anas acuta*) and Great Crested Grebes. In this period, Common Teals and Shovelers would be the species at high risk of being secondary cases (Table 2).

**Table 2:** Summary of estimates of the main species at high risk of being introducers and secondary cases for avian influenza viruses in the Ebre Delta (Spain) in 2006 and 2007.

Period of study	Proportions of main species at high-riskof being primary cases	Proportions of main species at high-risk of being secondary cases	
		With a R <1	with a R >1
Spring-Summer (March to September)	Mallard (68%)	Mallard (56%)	Mallard (38%)
		Coot (15%)	Coot (17%)
		Black-headed Gull (8%)	Black-headed Gull (9%)
	Common Tern (29%)	Common Tern (55%)	Common Tern (36%)
		Sandwich Tern (19%)	Sandwich Tern (19%)
		Audouin’s Gull (7%)	Audouin’s Gull (9%)
	Others species of the *Anatidae* and*Podicipedidae* families (3%)	Little Grebe (32%)	Mallard (32%)
		Mallard (22%)	Coot (17%)
		Shelduck (9%)	Red-crested Pochard (8%)
Autumn-Winter (October to February)	Mallard (57%)	Mallard (56%)	Mallard (33%)
		Herring Gull (9%)	Black-headed Gull (16%)
		Coot (8%)	Herring Gull (11%)
	Shoveler (21%)	Shoveler (45%)	Black-headed Gull (20%)
		Teal (15%)	Mallard (19%)
		Mallard (14%)	Shoveler (12%)
	Teal (16%)	Teal (50%)	Black-headed Gull (18%)
		Shoveler (14%)	Mallard (15%)
		Pintail (9%)	Teal (13%)
	Other species of the *Anatidae* and*Podicipedidae* families (6%)	Mallard (20%)	Mallard (22%)
		Shoveler (14%)	Black-headed Gull (16%)
		Great Crested Grebe (12%)	Coot (13%)

**Figure 3:**
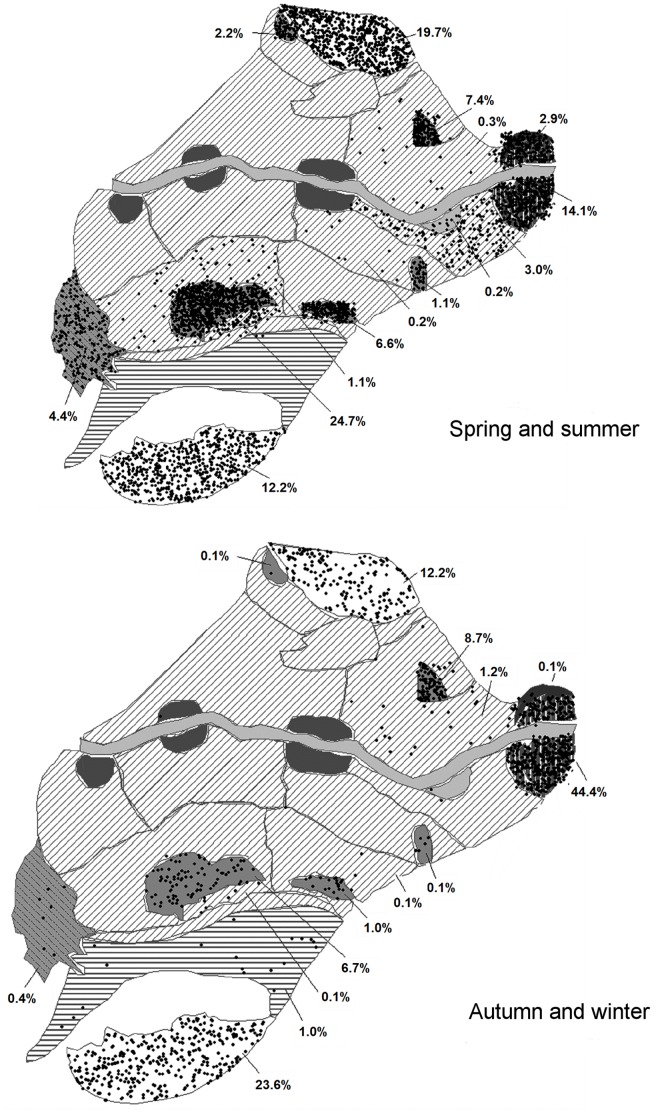
Maps of the geographical distribution of 10,000 incursions of infected wild birds in the spring and summer and in the autumn and winter.

As regards to the geographic distribution of AIV, the results showed that in the spring and summer, most of the AIV introductions would occur in lagoons, such as Encanyissada (B = 25) or Canal Vell (B = 9), followed by areas of sandy beaches and bays (i.e. Port Fangar (B = 22) or Punta de la Banya (B = 7)). Both ecosystems constituted the main spring and summer sites of the Ebre Delta. Whereas, in autumn and winter most of the AIV introductions would occur in the Island of Buda (B = 8) and Punta de la Banya (B = 7), where there were the highest densities of autumn and winter waterfowl (Figure 3).

**Figure 4:**
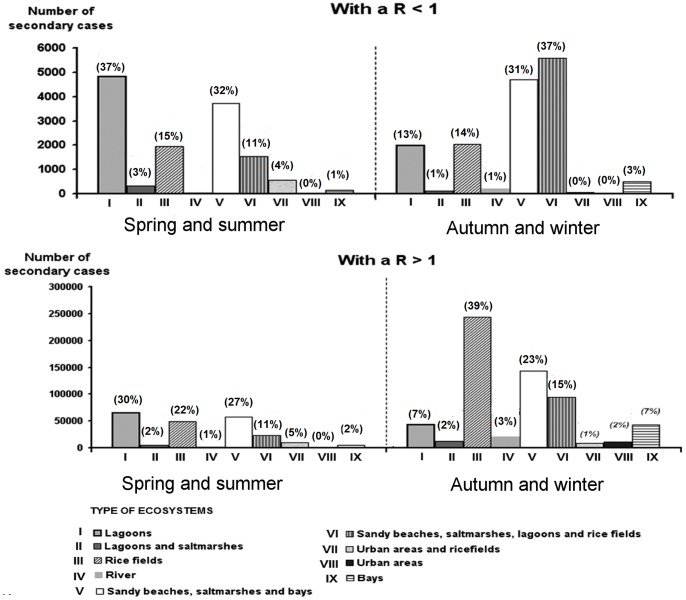
Geographical distribution of secondary cases according to type of ecosystem (in parenthesis the percentage of affected animals in each ecosystem).

Finally, the model predicted those areas at high risk of being affected by secondary infections (Figure 4). In spring and summer, the ecosystems at high risk would also be the lagoons and their surrounding areas and the area of Buda Island (B = 8), which is composed of sandy beaches, salt marshes, lagoons and rice fields. Whereas, in autumn and winter, the main high-risk areas affected by secondary cases would be the previous ones and rice fields.

### Sensitivity Analysis Results

**Figure 5:**
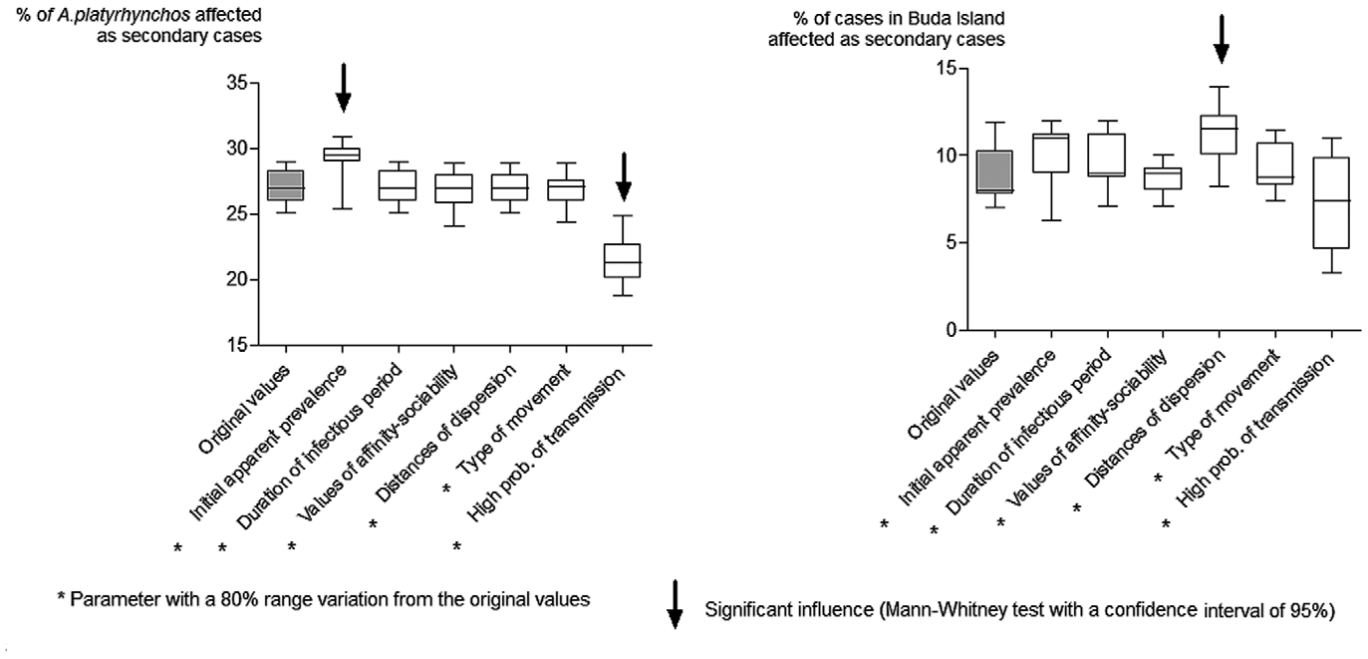
Results of the sensitivity analysis showing in the box plot graphs the influence of the main input parameters into the model in the spring and summer with a high R.

From this analysis, we observed that the output “infected species” (which includes both primary and secondary cases) was mainly influenced by the values of “initial apparent prevalence” and the “probability of transmission”. Whereas the areas at high risk were highly dependent on the distances of dispersion and the probability of transmission. The values of the level of significance of the Mann-Whitney test for each parameter and period are shown in Table S5. From these results, we observed that this influence was particularly important when the effective reproductive rate was higher than 1, which resulted in a remarkable increase in the number of cases located in areas not initially considered to be at high risk. As an example, we present the influence of the different parameters in the scenario for the spring and summer period considering an expected R higher than 1 (Figure 5).

## Discussion

Due to the complex interactions between diverse host and viral populations and the environments in which they cohabit, design, sampling and data interpretation constitute an important challenge for wildlife surveillance of AIV. In addition, these systems also face substantial logistical and financial constraints [11].

Our model was constructed to enhance the identification of those species, locations and periods that could give a better understanding of AI circulation in a specific area. We performed this model for the Ebro Delta as an example of the application of this approach. Based on its estimates, in the autumn and winter period, we would expect there to be a high risk of contacting AIV in dabbling ducks that inhabit areas of sandy beaches, salt marshes and rice fields and the bays of Buda Island and Punta de la Banya. These estimates were in agreement with the results obtained from the surveillance in Catalonia between 2006 and 2009 [15]. These findings could be explained by the high concentration of migrant waterfowl coming from Northern Europe, where the apparent prevalence of AIV seemed to be highest during the breeding season [18]. In contrast, in spring and summer, the type of ecosystem at high risk varied significantly depending on the species that acted as the primary case. During this period, lagoon areas might be mainly affected by AIV transmitted between Anseriformes; while the sandy beaches, salt marshes and bays would be affected by AIV transmitted between such Charadriiformes as Common Terns. In addition, from the comparison of different scenarios, we observed that if the transmission infection rates took higher values, other intermediate species, such as some species of Gull or Coot, might be more likely to be in contact with AIV. Consequently, if these species were infected, AIV could easily be dispersed in rice fields, where there are many poultry holdings and backyard flocks. These results suggest that Gulls and Coots in the Ebro Delta would be at high risk of acting as intermediaries due to their large number, broad distribution, and high degree of affinity with dabbling ducks and terns.

To predict the most likely avian subpopulations to be in contact with AIV on basis of ornithological and surveillance data, diverse assumptions about the mechanism of transmission, dynamics of avian populations, and possible states of infection were made. Next, we discuss the relevance of these assumptions with regard to the results and their interpretation.

Previous experimental studies and models were focused on the study of the main parameters of AI infection and the transmission dynamics of different subtypes among individual birds, mainly in Anseriformes [27]–[31].The model by Hénaux et al. [27] provided insights into those periods in which transmission in wild birds may be higher for LPAI and HPAI. However, most of these estimates have not been studied for numerous species and circulating subtypes. In this first approach, we dealt with this lack of basic information on the process of AIV spreading among birds by assigning homogeneous AI infection rates according to different scenarios. Consequently, although this model allowed estimation of those avian subpopulations that are most likely to be in contact with AIV, the approach did not allow prediction of the evolution of the spread of AIV.

In our model, the avian population was represented as a closed population in each period, with no demographic changes due to migratory movements, breeding, or hunting. This assumption would not affect the estimates whenever the proportions of each species with respect to other species remained constant throughout the period.

The model simulated a process of infection using an SIS compartmental model in which individual birds could only be susceptible or infected (without considering exposed, dead, or recovered states). According to previous studies, AIV has a short eclipse phase as infected birds [28]–[29] and the mortality rate caused by AIV could be considered almost nil [27], [30]–[31]. Consequently, not including such states as exposed or dead should not constitute a serious constraint. On the other hand, some previous studies reported the existence of a relative protection against hemagglutinin homologous and heterologous reinfections in such avian species as Mallards [21], [25]. However, the uncertainty and variability associated to the immune response, dependent on several factors associated with both hosts and subtypes, prevented us from representing the recovered state. In this regard, we require further studies to improve the understanding of the spread of AIV.

Although some environmental factors (temperature, wind, rainfall or soil or anthropic disturbances) may affect AIV transmission [32]–[33], their influence was not taken into account in this preliminary approach.

The sensitivity analysis revealed that the initial apparent prevalence of high-risk species had a strong influence on the species infected. This parameter varies by species, season, year and place [13], [17]–[18], and therefore, it would be essential to update and introduce the available information in accordance with the period of study. An example of its influence is shown in the estimates obtained for Common Terns. According to the model, this species could play an important role as introducer and spreader in spring in summer. These estimates may be explained by the high prevalence of LPAIV detected in this species in 2006 and 2007, especially in some Polish and Italian regions [19]–[20]. From these results, we supported the importance of combining harmonized systems on a large and local scale with paired active and passive surveillance to collect data on the AIV circulating between wild birds and predict their introduction to other areas in accordance with previous surveillance data [19]–[20], [34]–[37].

In addition, the distance of dispersion also had a strong influence on the areas at high risk, and the total number of cases generated in an epidemic. This may be explained by an increase in the range of dispersion increasing the possibilities of contact between birds.

In conclusion, this study presents a complementary tool to previous studies to track those subpopulations at high risk of contacting AIV in a complex ecosystem on a locale scale and to optimize the allocation of the limited resources for AI surveillance. This study also indicates the need for further research on the identification of host factors to determine the interspecies transmission of AIV.

## Supporting Information

Table S1 **Input values of the probabilities of transmission for the different scenarios simulated.**
(DOC)

Table S2 Probability of contact in accordance with the degree of affinity between an infected α* species and another α species (values assigned by expert opinion).
(DOC)

Table S3 **Probability of long range dispersion from r_α,i_ to position z_α,i_ by each species during each period of study (data obtained by expert opinion).**
(DOC)

Table S4 Description and notation of the inputs of the model.
(DOC)

Table S5 **Values of the level of significance of the Mann-Whitney test for each parameter and scenario obtained through the sensitivity analysis.**
(DOC)
