# Supplementary material for: Model to Track Wild Birds for Avian Influenza by Means of Population Dynamics and Surveillance Information
Source: PLoS One. 2012 Aug 30;7(8):e44354. doi: 10.1371/journal.pone.0044354 (PMC3431374; doi:10.1371/journal.pone.0044354)
Supplement: Table S1 — Input values of the probabilities of transmission for the different scenarios simulated. (DOC) [file pone.0044354.s001.doc]

|  | **Expected R < 1** | | **Expected R > 1** | |
| --- | --- | --- | --- | --- |
|  | **For species at high risk (α^R^)** | **For intermediate risk species (α^B^)** | **For species at high risk (α^R^)** | **For intermediate risk species (α^B^)** |
| P(α_i_* -> α_i_) | 0.4 | 0.3 | 0.8 | 0.6 |
| P(α_i_* -> α_j_) | 0.2 | 0.15 | 0.4 | 0.3 |

P (α_i_^*^ -> α_i_) : probability of transmission from an infectious bird when it contacts the same species, and P(α_i_ ^*^-> α_j_) when it contacts a different species.

Values assumed by the modellers considering two scenarios with different forces of infection: 1.a self-extinguishing epidemic and 2.a scenario with the development of an epidemic.
