# Supplementary material for: Model to Track Wild Birds for Avian Influenza by Means of Population Dynamics and Surveillance Information
Source: PLoS One. 2012 Aug 30;7(8):e44354. doi: 10.1371/journal.pone.0044354 (PMC3431374; doi:10.1371/journal.pone.0044354)
Supplement: Table S2 — Probability of contact in accordance with the degree of affinity between an infected α* species and another α species (values assigned by expert opinion). (DOC) [file pone.0044354.s002.doc]

| **Probability of contact in accordance with the degree of affinity between an infected α* species and another α species** | **Values of a_α*,α_** |
| --- | --- |
| Same species | 0.8 -1 |
| Those species that share habitat and feeding or nesting group and show high gregarious behaviour | 0.8 |
| Those species that share habitat but not group, and do not have high gregarious behaviour | 0.4 |
| Those species that have high gregarious behaviour and sporadically share habitat | 0.2 |
| Those species that do not have gregarious behaviour and sporadically share habitat and feeding | 0.05 |
| Those species that do not have gregarious behaviour and rarely share habitat and feeding | 0.02 |
| No relationship | 0.01 |
