# Supplementary material for: Model to Track Wild Birds for Avian Influenza by Means of Population Dynamics and Surveillance Information
Source: PLoS One. 2012 Aug 30;7(8):e44354. doi: 10.1371/journal.pone.0044354 (PMC3431374; doi:10.1371/journal.pone.0044354)
Supplement: Table S3 — Probability of long range dispersion from rα,i to position zα,i by each species during each period of study (data obtained by expert opinion). (DOC) [file pone.0044354.s003.doc]

|  | **Probability of dispersion movements of each species to other**  **positions located within the following ranges of distances** | | | | | | | |
| --- | --- | --- | --- | --- | --- | --- | --- | --- |
|  | **In Spring and Summer** | | | | **In Autumn and Winter** | | | |
| **Family: Species** | **>0.2-1km** | **>1-5km** | **>5-10km** | **>10km** | **>0.2-1km** | **>1- 5km** | **>5-10km** | **>10km** |
| *Anatidae:* Pintail.Teal, Wigeon, Tufted Duck | - | - | - | - | 0.15 | 0.65 | 0.1 | 0.1 |
| *Anatidae:* Mallard, Gadwall, Pochard, Red-crested Pochard | 0.5 | 0.4 | 0.1 | 0 |  |  |  |  |
| *Phoenicopteridae:* Greater Flamingo | 0 | 0.5 | 0.25 | 0.25 |  |  |  |  |
| *Anatidae:* Shoveler | 0.8 | 0.2 | 0 | 0 |  |  |  |  |
| *Anatidae:* Shelduck |  |  |  |  | 0.8 | 0.1 | 0.1 | 0 |
| *Recurvirostridae:* Black-winged Stilt, Avocet  *Scolopacidae:* Redchnack |  |  |  |  |  |  |  |  |
| *Podicipedidae:* Great Crested Greber, Little Grebe  *Rallidae:* Coot | 0.9 | 0.1 | 0 | 0 |  |  |  |  |
| *Charadriidae:* Kentish Plover, Grey Plover | - | - | - | - |  |  |  |  |
| *Scolopacidae:* Black-tailed, Godwit.Ruff |  |  |  |  |  |  |  |  |
| *Glareolidae:* Whiskered Tern | 0.1 | 0.8 | 0.1 | 0 |  |  |  |  |
| *Ardeidae:* Grey Heron |  |  |  |  | 0.1 | 0.8 | 0.1 | 0 |
| *Laridae:* Slender-billed Gull | 0 | 0.7 | 0.2 | 0.1 | 0 | 0.8 | 0.1 | 0.1 |
| *Laridae:* Herring Gull | 1 | 0 | 0 | 0 |  |  |  |  |
| *Laridae:* Audouin's Gull | 0 | 0.1 | 0.2 | 0.7 |  |  |  |  |
| *Laridae:* Black-headed Gull |  |  |  |  | 0 | 0 | 0.75 | 0.25 |
| *Sternidae:* Little Tern, Common Tern, Gud-billed Tern, Sandwich Tern | 0 | 0.7 | 0.3 | 0 | - | - | - | - |
| *Glareolidae:* Collared pratincole |  |  |  |  | 1 | 0 | 0 | 0 |
| *Charadriidae:* Lapwing | - | - | - | - | 0.1 | 0.2 | 0.7 | 0 |
| *Anatidae:* Greylag Goose |  |  |  |  | 0.9 | 0.1 | 0 | 0 |
