# Supplementary material for: Model to Track Wild Birds for Avian Influenza by Means of Population Dynamics and Surveillance Information
Source: PLoS One. 2012 Aug 30;7(8):e44354. doi: 10.1371/journal.pone.0044354 (PMC3431374; doi:10.1371/journal.pone.0044354)
Supplement: Table S4 — Description and notation of the inputs of the model. (DOC) [file pone.0044354.s004.doc]

| **Description of the main inputs** | **Notation** |
| --- | --- |
| Total population of wild birds considered in each period of study | P (P= Pw, Pb) |
| Species considered | α(α= 1, 2,…, d) |
| Census of each species in the Ebre Delta | Nα |
| Species at high risk | α^R^ |
| Intermediate species | α^B^ |
| Areas defined in the Ebre Delta according to type of ecosystem and location | B (B = 1, 2,…, 27) |
| Number of birds of the α species in the B polygon | n_B,α_ |
| Apparent Prevalence in European Countries detected between 2006-2007 | Prev_α_ |
| Average number of secondary cases | R |
| Infectious period | τ_α,i_ |
| Degree of affinity between the infected species and susceptible species | a_α*,α_ |
| Time step used in the model | Δt |
| Probability of movement within the time step that the infected bird has in a determined location | φ_α,i_ |
| Movement to a long range dispersion by species | f(z_α_|r_α_) |

|  | **% of secondary cases as Mallards** | | | | **% of birds in Buda Island infected as secondary cases** | | | |
| --- | --- | --- | --- | --- | --- | --- | --- | --- |
|  | **Expected R lower than 1** | | **Expected R higher than 1** | | **Expected R lower than 1** | | **Expected R higher than 1** | |
| **Parameters** | **Spring and summer** | **Autumn and winter** | **Spring and summer** | **Autumn and winter** | **Spring and summer** | **Autumn and winter** | **Spring and summer** | **Autumn and winter** |
| **Initial apparent prevalence** | 0.02 * | 0.00 * | 0.01 * | 0.00 * | 0.34 | 0.00 * | 0.08 | 0.00 * |
| **Duration of infectious period** | 0.30 | 0.09 | 0.88 | 0.65 | 1.00 | 0.27 | 0.26 | 0.60 |
| **Values of affinity -sociability** | 0.61 | 0.09 | 0.79 | 0.41 | 0.18 | 0.25 | 0.78 | 0.12 |
| **Distances of dispersion** | 0.94 | 0.67 | 0.79 | 0.07 | 0.88 | 0.08 | 0.01 * | 0.00 * |
| **Type of movement** | 0.68 | 0.09 | 0.97 | 0.67 | 0.97 | 0.47 | 0.31 | 0.42 |
| **Probability of transmission** | 0.07 | 0.21 | 0.00 * | 0.00 * | 0.09 | 0.07 | 0.31 | 0.00 * |

* p-value lower than 0.05

Table S5. Values of the level of significance of the Mann-Whitney test for each parameter and scenario obtained through the sensitivity analysis.

**SUPPORTING INFORMATION LEGENDS**

Table S1. Input values of the probabilities of transmission for the different scenarios simulated.

Table S2. Probability of contact in accordance with the degree of affinity between an infected α* species and another α species (values assigned by expert opinion).

Table S3. Probability of long range dispersion from r_α,i_ to position z_α,i_ by each species during each period of study (data obtained by expert opinion).

Table S4. Description and notation of the inputs of the model.

Table S5. Values of the level of significance of the Mann-Whitney test for each parameter and scenario obtained through the sensitivity analysis.
