# Supplementary material for: Model to Track Wild Birds for Avian Influenza by Means of Population Dynamics and Surveillance Information
Source: PLoS One. 2012 Aug 30;7(8):e44354. doi: 10.1371/journal.pone.0044354 (PMC3431374; doi:10.1371/journal.pone.0044354)
Supplement: Table S5 — Values of the level of significance of the Mann-Whitney test for each parameter and scenario obtained through the sensitivity analysis. (DOC) [file pone.0044354.s005.doc]

|  | **% of secondary cases as Mallards** | | | | **% of birds in Buda Island infected as secondary cases** | | | |
| --- | --- | --- | --- | --- | --- | --- | --- | --- |
|  | **Expected R lower than 1** | | **Expected R higher than 1** | | **Expected R lower than 1** | | **Expected R higher than 1** | |
| **Parameters** | **Spring and summer** | **Autumn and winter** | **Spring and summer** | **Autumn and winter** | **Spring and summer** | **Autumn and winter** | **Spring and summer** | **Autumn and winter** |
| **Initial apparent prevalence** | 0.02 * | 0.00 * | 0.01 * | 0.00 * | 0.34 | 0.00 * | 0.08 | 0.00 * |
| **Duration of infectious period** | 0.30 | 0.09 | 0.88 | 0.65 | 1.00 | 0.27 | 0.26 | 0.60 |
| **Values of affinity -sociability** | 0.61 | 0.09 | 0.79 | 0.41 | 0.18 | 0.25 | 0.78 | 0.12 |
| **Distances of dispersion** | 0.94 | 0.67 | 0.79 | 0.07 | 0.88 | 0.08 | 0.01 * | 0.00 * |
| **Type of movement** | 0.68 | 0.09 | 0.97 | 0.67 | 0.97 | 0.47 | 0.31 | 0.42 |
| **Probability of transmission** | 0.07 | 0.21 | 0.00 * | 0.00 * | 0.09 | 0.07 | 0.31 | 0.00 * |

* p-value lower than 0.05
